# A Cost‐Effective Nonaqueous Reversed‐Phase High‐Performance Liquid Chromatography Method to Measure Vitamin D3 in Hen's Egg Yolk

**DOI:** 10.1002/jssc.70087

**Published:** 2025-01-23

**Authors:** Ina Varfaj, Alice Cartoni Mancinelli, Anna Migni, Laura Mercolini, Cesare Castellini, Francesco Galli, Desirée Bartolini, Roccaldo Sardella

**Affiliations:** ^1^ Department of Pharmaceutical Sciences University of Perugia Perugia Italy; ^2^ Department of Agricultural Food and Environmental Sciences University of Perugia Perugia Italy; ^3^ Department of Pharmacy and Biotechnology (FaBiT) Alma Mater Studiorum‐University of Bologna Bologna Italy

**Keywords:** HPLC column screening, method validation, quantitative analysis, silanol activity, spectrophotometric detection

## Abstract

The objective of this study is to develop an HPLC‐UV method for the cost‐effective and quantitative determination of vitamin D3 in food, even in the presence of vitamin D2, with a specific focus on egg yolk. During method development, the performance of three stationary phases in resolving the peak of vitamin D2 from that of vitamin D3 was investigated. The physicochemical properties of these phases differed particularly in the extent of hydrophobicity and silanophilic activity, including a GraceSmart RP C18 column without silanol endcapping, a Robusta RP C18 column with silanol endcapping, and a Waters Xbridge RP C18 column *with ethylene‐bridged hybrid (BEH) particle technology*. The Xbridge C18 stationary phase exhibited the most favorable performance, leading to an *R*_S_ of 1.6 under the following nonaqueous reversed‐phase (NARP) experimental conditions: mobile phase, acetonitrile, methanol, and trifluoroacetic acid in a (99/1/0.1, v/v/v) ratio; column temperature, 15°C. The developed chromatographic method does not require preanalytical purification steps and is also compatible with mass spectrometry. *The identity of the vitamin D3 peak observed in the HPLC analysis was verified via GC–MS. The NARP‐HPLC‐UV method was partially validated*, demonstrating satisfactory linearity, precision, accuracy, limit of quantification, and robustness. The HPLC method was then successfully applied to the analysis of real egg yolk samples, revealing average concentrations of vitamin D3 of 4–5 µg/g of wet weight sample.

## 1 Introduction

Vitamin D includes a group of fat‐soluble compounds that function both as a *micronutrient* and as a hormone, playing an essential role in human development and overall health. Although primarily recognized for its role in maintaining calcium and phosphorus homeostasis [1], vitamin D is also associated with a variety of other health advantages [2, 3, 4, 5, 6].

**FIGURE 1:**
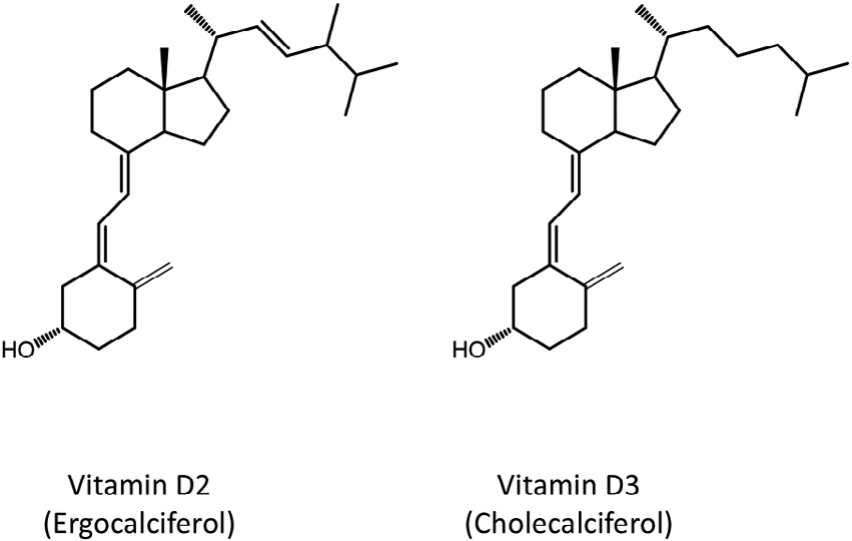
Structure of vitamin D2 (ergocalciferol) and vitamin D3 (cholecalciferol).

Although it can be synthesized endogenously, dietary intake is essential to maintain adequate levels, highlighting its crucial role in human physiology. In nature, vitamin D occurs in two principal forms: ergocalciferol (commonly referred to as vitamin D2) and cholecalciferol (commonly referred to as vitamin D3) (Figure 1).

Ergocalciferol is sourced from food of plant origin, particularly from mushrooms and yeasts, whereas cholecalciferol is sourced from skin exposure to ultraviolet B radiation (UVB, usually sunlight) on its precursor, 7‐dehydrocholesterol, in skin and mainly food of animal origin [7]. In general, the vitamin D content in food depends on its fat content, the diet fed to the animals, and the food's exposure to UVB light [8].

The status of vitamin D in the body is contingent upon both dietary intake and endogenous synthesis, the latter of which is influenced significantly by exposure to sunlight [9]. In humans, about 80% of the body's vitamin D comes from UVB exposure in the skin, while diet provides much less [10].

It is important to note that sunlight exposure alone is often inadequate to fulfill vitamin D requirements, particularly given the widespread prevalence of indoor lifestyles. Additionally, seasonal fluctuations in sunlight intensity may influence the endogenous synthesis of vitamin D in both humans and animals [11, 12].

In contrast to vitamin D synthesized through sun exposure, dietary vitamin D is available year‐round, providing a reliable source for individuals with limited sunlight exposure. Consequently, the demand for vitamin D from dietary sources is on the rise. Within daily nutrition, eggs represent an excellent natural source of vitamin D [13, 14, 15, 16].

Regarding animal species, domestic poultry like chickens (*Gallus gallus*) and turkeys (*Meleagris gallopavo*) play an increasingly important role in meeting the nutritional needs of the global population [17]. Chickens, in particular, are among the most valuable sources of vitamin D due to their production of meat and eggs for human consumption.

Numerous studies have been conducted to increase the vitamin D3 content in hen eggs, focusing on dietary modifications to enrich this fat‐soluble vitamin [14, 18]. The analytical detection and quantification of vitamin D2 and vitamin D3 in hen eggs continue to present significant challenges. The extremely low concentrations of these compounds (on the order of a few micrograms per 100 g of food matrix) necessitate *sensitive* detection amidst the presence of other abundant compounds, such as fats, proteins, and other fat‐soluble vitamins. Currently, the coupling of high‐performance liquid chromatography (HPLC) to tandem mass spectrometry (HPLC‐MS/MS) is frequently reported in the literature and is considered the most appropriate technique for accurately quantifying vitamin D in complex food matrices [19, 20]. However, the high costs associated with HPLC‐MS/MS systems, coupled with the substantial technical expertise required, continue to hinder their routine application in many food control laboratories. Consequently, traditional HPLC‐UV methods are still more commonly utilized for this purpose in the current scenario.

To date, only a limited number of HPLC methods have been developed for the accurate quantification of vitamin D3 in egg yolk. Jackson, Shelton, and Frier were among the first to successfully apply HPLC to determine vitamin D3 levels in various food matrices, including eggs, butter, milk, and cheese [21]. Their method involved vitamin D2 as an internal standard. Vitamins D2 and D3 were separated using a reversed‐phase HPLC (RP‐HPLC) method, employing a C22 stationary phase and an aqueous mobile phase with methanol (MeOH) as the organic modifier. It is important to note that the method developed by Jackson, Shelton, and Frier exhibited limited sensitivity, necessitating the use of up to 50 g of lyophilized egg mass for vitamin analysis. Furthermore, baseline separation (resolution) between the two peaks was not achieved, and the accuracy of the method was contingent on how the internal standard was introduced into the sample, as well as the consistency of the ratio of vitamin D2 to D3 throughout the analytical process.

Takeuchi et al. [22, 23] employed a nonaqueous reversed‐phase HPLC (NARP‐HPLC) method for the analysis of vitamins D2 and D3 in egg yolk extracts utilizing a C18 stationary phase and an acetonitrile (ACN)/MeOH‐based eluent. The analytical procedure was preceded by a preparative NARP‐HPLC analysis that facilitated the isolation of the vitamin D‐containing fraction from the examined food matrix. However, the proposed method faced challenges in accurately quantifying vitamin D3 in the food matrix due to the lack of resolution between the internal standard vitamin D2 and the target vitamin D3. Furthermore, significant vitamin losses might occur due to pre‐analytical saponification and purification processes [22].

A sensitive and reliable HPLC method was developed by Mattila et al. [24]. The method for quantifying vitamin D3 was designed to incorporate saponification, extraction, and optimization of purification procedures. The study indicated the use of semipreparative HPLC under normal phase (NP) conditions. Subsequently, an analytical HPLC method was developed that efficiently separates both vitamins D2 and D3 for quantification using a C18 column with a polar organic mobile phase composed of MeOH and water. Despite potential vitamin losses during purifications, the overall average recovery for both vitamins was estimated at 70%, potentially affecting measurement accuracy. Nonetheless, from a purely chromatographic perspective, the method proposed by Mattila et al. remains an effective approach for determining vitamin D3 content in egg yolks.

The present study is within this framework and aims to develop an HPLC‐UV method for the quantitative analysis of vitamin D3 in egg yolk samples, also in the presence of vitamin D2 derived from the animal's diet. A key advantage of this method is its ability to clearly resolve vitamin D2 from vitamin D3, ensuring a satisfactory chromatographic peak separation. *The proposed HPLC method, which is also easily adapted for use with MS systems, is designed to enable the facile quantification of both vitamin D2 and vitamin D3 after easy‐to‐perform saponification and extraction steps while avoiding preliminary chromatography‐based purification procedures*. This approach not only streamlines the analytical workflow but also improves the reliability of the quantification process.

## 2 Materials and Methods

### 2.1 Chemicals, Reagents, and Egg Yolk Samples

All the reagents and solvents used in the study were of analytical grade. ACN, MeOH, ethanol (EtOH), water, trifluoroacetic acid (TFA), isopropyl alcohol (IPA), *n*‐hexane, ethyl acetate, potassium hydroxide (KOH), toluene, the derivatizing reagent *N*,*O*‐bis‐(trimethylsilyl) trifluoroacetamide (BSTFA, 99.4%), pyridine, and vitamin D3 (cholecalciferol) were purchased from Merck Life Science (Merck KGaA, Darmstadt, Germany). Vitamin D2 (ergocalciferol) was purchased from Cayman Chemical (Ann Arbor, Michigan, USA). Water for HPLC analysis was purified with a New Human Power I Scholar water purification system (Human Corporation, Seoul, Korea). Hens' eggs were standard eggs (weight category 60 g, reared in a barn) bought in a local supermarket.

### 2.2 Extraction From Egg Yolk

*The method for the quantification of vitamin D3 in the lyophilized egg yolks was based on existing protocols* [22, 23, 24, 25], *with only marginal modifications*.

In accordance with Okano [23], an aliquot of 0.5 g of a sample of the homogenized egg yolk was weighed and subjected to extraction. The sample was manually homogenized to reduce the size of the original material and increase the contact surface area of the weighed powder of egg yolk with the extraction solvent. *The saponification procedure was conducted at room temperature for 2 h*, *keeping the mixture under stirring with a magnetic stirrer in a flask covered with aluminum foil*. Accordingly, to the *weighed* sample, 5 mL of a previously prepared solution consisting of 15% KOH (w/v) dissolved in plain EtOH was added, and the saponification procedure was conducted at room temperature for 2 h under stirring as described above.

Subsequently, the contents of the saponification flask were transferred into a 50 mL flask and extracted with 5 mL of a mixture of *n*‐hexane and ethyl acetate (95/5, v/v), according to [26], and vortexed for 5 min. The upper phase was transferred to a separate 15 mL falcon tube and dried under a gentle nitrogen stream. The extraction procedure was repeated twice, and the samples were combined in a unique flask.

To the dried extract, a volume of 2 mL of EtOH was added. Subsequently, two aliquots, each of 1 mL, were transferred into two separate vials. The two ethanolic solutions were further dried under a gentle nitrogen stream and stored at −20°C before the HPLC‐PDA analysis. For the analysis, the yellow residue was redissolved in 200 µL of pure EtOH.

### 2.3 Instrumentation and Chromatographic Conditions

The HPLC study was performed on a Waters ALLIANCE 2695 Separations Module system equipped with a quaternary, low‐pressure mixing pump, in‐line vacuum degassing, an autosampler with a maximum capacity of 120 vials, and a column heater/cooler. The system was equipped with a photodiode array (PDA) detector (Waters 2996), while data management was made by means of Waters Millennium32 Software. A GraceSmart RP C18 column (250 mm × 4.6 mm i.d., 5 µm, 100 Å pore size, Grace, Sedriano, Italy), a Robusta RP C18 column (250 mm × 4.6 mm i.d., 5 µm, 110 Å pore size, Sepachrom Srl, Italy), and a Waters Xbridge RP C18 column (150 × 4.6 mm, 5 µm i.d.; 130 Å pore size; from Waters Corporation, Milford, MA, USA) were comparatively evaluated as the analytical columns for the HPLC analysis for the food samples. The Waters Xbridge RP18 column resulted to be the optimal choice for the HPLC study. The UV detection wavelength was set at 265 nm, while the optimal flow rate was set at 0.5 mL/min. Column and sample rack temperatures were set both at 15°C. Injected samples were *dissolved* in plain EtOH. The injection volume was 20 µL for all the analyses. The optimal mobile phase composition was found to be ACN/MeOH (99/1, v/v) with 0.1% TFA (v/v).

The quantitative HPLC analysis was performed using calibration curves properly built up by plotting the concentrations of seven calibration standard solutions versus the corresponding peak areas. The tested linearity concentration ranges were 0.25–2 µg/mL. A range of concentrations between 50% and 150% of the expected/theoretical vitamin D3 concentration was used. The coefficient of determination (*R* ^2^) resulted in a value higher than 0.9991. The chromatographic method was validated according to a research‐type protocol.

As far as the GC–MS analysis is concerned, the residue obtained after extraction was dissolved into 50 µL of anhydrous pyridine and silylated at 65°C for 90 min with 50 µL of BSTFA. In Figure S1, the reaction scheme for vitamin D3 is shown.

The GC–MS analysis was performed with a GC–MS system consisting of a GC 7890A gas chromatograph equipped with a VF–5 ms capillary column (15 m × 0.15 mm internal diameter, 0.15 µm film thickness) + 5 m EZ‐Guard (Chrompack Middelburg, Netherlands), coupled to a VL‐MSD triple‐axis detector 5975C (Agilent Technologies Inc., Santa Clara, CA, USA).

Helium was used as a carrier gas with a flow of 1.0 mL/min, and the injector operated at 300°C in pulsed splitless mode (pressure 50 psi). The oven temperature was as follows: from 120°C (held for 1.5 min) to 240°C at 113°C/min, then to 280°C at 8.5°C/min (kept for 1.5 min), to 350°C at the rate of 28.3°C/min, and finally, the system was maintained under isothermal conditions for 3 min. Transfer line, ion source, and quadrupole temperatures were set at 300°C, 240°C, and 150°C, respectively. For D3 analysis, the injection volume was 0.5 µL, and the mass spectrometer operated in selected ion monitoring mode (EI+ SIM) at 70 eV: 325 and 351 *m*/*z*.

## 3 Results and Discussion

### 3.1 NARP‐HPLC‐UV Method Optimization

#### 3.1.1 Mobile Phase Composition and Type of Reversed‐Phase C18 Column

The initial phase of HPLC method development involved the selection of a stationary phase that facilitated the baseline separation (resolution) of peaks associated with vitamin D2 and vitamin D3. This portion of the study investigated three stationary phases, each characterized by distinct physicochemical properties, particularly with regard to the extent of hydrophobicity and silanophilic activity [27]. These attributes significantly influence the RP mechanism and the overall quality of chromatographic performance [28].

To determine the optimal conditions for the separation and quantification of vitamin D2 and vitamin D3, two commercially available and relatively conventional RP C18 columns were initially assessed in a comparative manner: a Robusta C18 (endcapped alkyl‐bonded stationary phase) and a Grace Smart C18 (non‐endcapped alkyl‐bonded stationary phase). For the comparative analysis, identical chromatographic conditions were ensured in terms of column temperature, mobile phase composition, and flow rate.

Both columns are well‐suited for regular and intensive use, providing high performance and exceptional longevity. It is crucial to emphasize that due to the bonding procedures employed to produce conventional silica‐based RP C18 packings, some residual free silanol groups may remain unreacted on the silica surface. These groups can interact with polar compounds, often adversely affecting the kinetic characteristics of the chromatographic process [29]. Endcapping the bonded phase aims to mitigate these secondary interactions, thus enhancing the mass transfer process and improving peak shape. Furthermore, the activation of a dual retention mechanism can also negatively affect the system selectivity, influencing the thermodynamic properties of the chromatographic process [30].

According to literature findings, the HPLC analysis of vitamin D3 has been effectively conducted using binary and ternary eluent systems, predominantly comprising ACN in conjunction with lower proportions of MeOH and water [22–24, 31–33]. In light of this, a ternary mixture consisting of ACN, MeOH, and water in a volumetric ratio of (95/2.5/2.5, v/v/v) was initially utilized, with the analysis performed at a column temperature of 30°C. Despite the screening mobile phase yielding a resolution (*R*_S_) between vitamin D2 and vitamin D3 peaks of less than 1.5, the use of the Robusta C18 column demonstrated superior kinetic performance compared to the Grace Smart C18 *column* (data not shown). *Besides the beneficial effect of the endcapping procedure in the Robusta C18 column, its superiority over the Grace Smart C18 can additionally be ascribed to the higher carbon load in the former (17.8%) over the latter (10%) column*. Thus, Robusta C18 was therefore selected for subsequent mobile phase optimization.

The results obtained are in accordance with the hydrophobic subtraction model (HSM) proposed by Snyder [27, 34], which quantitatively characterizes the chromatographic selectivity of RP‐HPLC columns. The Robusta RP C18 column exhibits intermediate properties regarding both the H‐bond acidity value and the interaction capabilities with ionized solute molecules at pH 7 [35]. In contrast, the Grace Smart RP C18 column displays higher values of H‐bond acidity and interaction capability with ionized solute molecules at the same pH [35].

To evaluate the advantages of employing isocratic conditions versus running gradient profiles, several gradient programs were systematically tested. The chromatographic performance was unaffected by the varying gradient conditions (data not shown); consequently, although aware that gradient helps to clean the column, all subsequent analyses were conducted under isocratic conditions. Thus, a one‐variable‐at‐a‐time (OVAT) experimental design was implemented to optimize the resolution of the two target compounds within a reasonable analysis timeframe. To achieve baseline separation (*R*_S_ ≥ 1.5) while maintaining ACN content at 95% (v/v), the remaining 5% of the mobile phase was comprised entirely of MeOH, resulting in a binary eluent system. As anticipated (Table 1, Entries a and b), the compounds eluted earlier and are consistent with a typical RP mechanism. While baseline separation was not attained with this binary system, an observable improvement in peak shape was noted (data not shown). Consequently, additional binary eluents with a significant presence of ACN were evaluated. Reducing the MeOH content from 5% to 1% (v/v) maintained consistent chromatographic performance in terms of peak shape while yielding increased retention times with unchanged selectivity (Table 1, Entry c). This suggests that the alcoholic additive enhances the solubility of the compounds, thereby reducing their residence time within the column. Nevertheless, the mobile phase containing lower MeOH content was preferred, as higher proportions of ACN may enhance UV detection sensitivity while concurrently minimizing eluent viscosity.

**TABLE 1:** Chromatographic performance obtained in the analysis of vitamin D2 and D_3_. *k*_1_ and *k*_2_ are, respectively, the retention factors of vitamin D_2_ and vitamin D_3_; *α* is the separation factor of vitamin D_2_ and vitamin D_3_; *R*_S_ is the resolution factor of vitamin D_2_ and vitamin D_3_. In all the cases, the elution order (EO) is: vitamin D_2_ < vitamin D_3_.

Entry	Column	Mobile phase	Chromatographic parameters					
			*k*_1_	*k*_2_	*α*	*R*_S_	Flow rate (mL/min)	Column *T* (°C)
a	Robusta	ACN/MeOH/water 95/2.5/2.5 (v/v/v)	5.50	5.87	1.07	<0.80^a^	1.0	30
b		ACN/MeOH 95/5 (v/v)	4.09	4.36	1.07	<0.80^a^	1.0	30
c		ACN/MeOH 99/1 (v/v)	4.52	4.85	1.07	<0.80^a^	1.0	30
d		ACN/EtOH 99/1 (v/v)	4.12	4.4	1.07	<0.80^a^	1.0	30
e		ACN/MeOH/IPA 98/1/1 (v/v/v)	3.93	4.19	1.07	<0.80^a^	1.0	30
f	Xbridge	ACN/MeOH 99/1 (v/v)	0.76	0.84	1.05	0.80	1.0	30
g		ACN/MeOH 99/1 (v/v)	1.99	2.18	1.09	1.45	1.0	15
h		ACN/MeOH/TFA 99/1/0.1 (v/v/v)	2.07	2.27	1.10	1.45	1.0	15
i		ACN/MeOH/TFA 99/1/0.1 (v/v/v)	5.15	5.54	1.08	1.60	0.5	15

^a^ Not calculated by the HPLC system data management software.

Considering that the interactions between the analytes and the stationary phase were determined to be influenced by the alcohol content, it was deemed pertinent to investigate additional “alcoholic modifiers.” Consequently, EtOH was employed alone as a replacement for MeOH, while IPA was utilized in conjunction with MeOH (refer to Table 1, Entries d and e). These modifications to the eluent did not enhance chromatographic performance; instead, they resulted in a decreased retention of analytes due to their increased solubility in these systems.

Since baseline separation was not achieved with the Robusta C18 column, an alternative C18 column was subsequently evaluated with the objective of improving the *R*_S_ between the peaks related to vitamin D2 and vitamin D3. For this purpose, the Waters XBridge C18 phase (details provided in Section 2.3) *was selected*, as this phase is manufactured using a distinct strategy aimed at mitigating the adverse effects of free silanols on overall chromatographic performance.

The XBridge C18 column is designed to optimize efficiency, chemical stability, and robustness. *In terms of materials, the XBridge C18 stationary phase has a comparable carbon load (17%) to Robusta RP C18* and employs bridge‐ethyl hybrid (BEH) technology, derived from two high‐purity monomers: tetraethoxysilane and bis(triethoxysilyl)ethane, which incorporates a preformed ethylene bridge. XBridge C18 columns feature a patented monofunctional silane whose embedded polar carbamate group bestows unique selectivity characteristics and superior peak shape, predominantly for, but not limited to, basic analytes [35].

The internal and surface hybrid groups confer distinctive chemical properties to this material, making it advantageous for separation applications. Utilizing the optimal eluent system with the XBridge C18 column significantly improved chromatographic performance compared to the Robusta C18 column, with analytes eluting at suitable retention times (see Table 1, Entry f). This represented the first condition under which the HPLC system data management software calculated an *R*_S_ value of 0.8.

#### 3.1.2 Effect of Column Temperature, Presence of an Acidic Additive, and Eluent Flow Rate

Column temperature can significantly influence chromatographic performance since analyte retention and column selectivity are thermodynamically governed parameters [36, 37, 38]. The variation in retention with temperature is often dependent on the characteristics of the analytes being investigated. Generally, an increase in column temperature leads to a decrease in analyte retention, while lower temperatures typically result in extended retention times.

In HPLC applications, elevated temperatures are utilized to reduce solvent viscosity and alleviate the backpressure associated with the use of smaller particle sizes and high flow rates. Adjusting the column temperature also favorably affects the kinetic properties of the chromatographic process [36, 39–42]. A higher column temperature generally accelerates the chromatographic process, potentially enhancing its efficiency. However, it is important to note that the percentage decrease in retention time is not uniform across all compounds in a sample mixture, and variations in peak separation are frequently observed. In these instances, the separation factors of the sample compounds may alter; *in most cases, increasing the column temperature results in reduced separation factors, sometimes accompanied by an increase in the resolution factor due to higher efficiency (higher plate numbers)*. This phenomenon is particularly noticeable when high separation values are achieved with excessively prolonged retention times. Consequently, raising the column temperature may serve as a viable strategy to shorten analyte retention, albeit at the cost of separation factor values.

Taking into account the aforementioned factors and the relatively modest separation between the peaks of interest, a column temperature of 15°C (as opposed to the initial value of 30°C) was evaluated, yielding an enhancement in the resolution factor (*R*_S_ from 0.8 to 1.45) at usable retention times (Table 1, Entry g).

The enhancement of this parameter can primarily be attributed to the increase in analyte retention and to the enhancement in column selectivity. Importantly, the reduction in column temperature did not adversely affect the system selectivity (Table 1, Entry g). Thus, the observed improvement in the resolution factor was fundamentally due to the extended retention experienced by the two compounds at the lower column temperature.

To achieve baseline separation, an acidic additive was introduced into the eluent and subsequently tested.

As an effective deprotonation suppressor for the residual underivatized silanols present on the surface of the stationary phase, 0.1% (v/v) TFA was incorporated into the mobile phase to mitigate peak broadening. With this additional eluent enhancement, chromatographic performance did not show any improvement at a flow rate of 1.0 mL/min (Table 1, Entry h). However, complete peak resolution was achieved when the flow rate was reduced to 0.5 mL/min, resulting in a resolution factor of 1.6 for the peaks associated with vitamin D2 and D3 (Table 1, Entry i). *The same improvement was not achieved without the acidic additive that, therefore, was included in the eluent system*. It is plausible to hypothesize that TFA acted in a way to mitigate the activation of secondary interaction mechanisms.

The observed enhancement in the resolution factor concomitant with a reduction in eluent flow rate strongly indicates the significant influence of the mass transfer term (the C‐term) in the Van Deemter equation on column efficiency [42].

**FIGURE 2:**
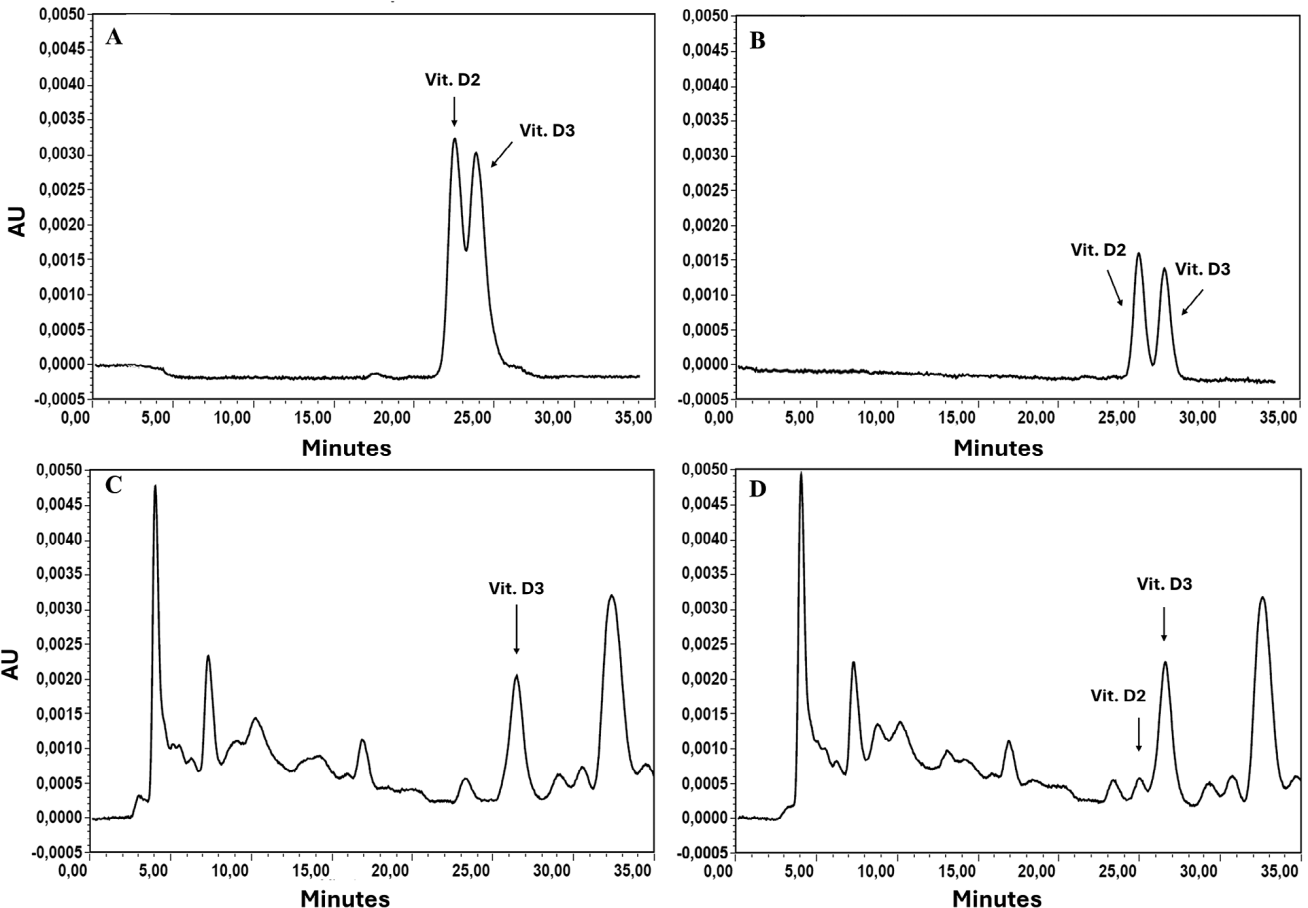
HPLC chromatogram of (A) a standard mixture of vitamin D2 and vitamin D3 obtained in the best chromatographic conditions with Robusta RP C18 stationary phase using conditions in Table 1, Entry c; (B) a standard mixture of vitamin D2 and vitamin D3 under the best chromatographic conditions obtained with Xbridge RP C18 stationary phase using the chromatographic conditions reported in Table 1, Entry i; (C) an exemplary extract from an egg yolk sample, obtained applying the chromatographic conditions reported in Table 1, Entry i; (D) a real extract from an egg yolk sample spiked with a solution containing both vitamins D2 and D3, using the final optimized chromatographic conditions reported in Table 1, Entry i.

Figure 2 presents the optimal chromatograms obtained using the Robusta C18 column (Figure 2A) and the XBridge C18 column (Figure 2B). For both analyses, the optimal conditions identified for the respective columns were employed.

### 3.2 Application of the Optimized NARP‐HPLC‐UV Method to the Analysis of Real Samples

It has been documented that esterified vitamin D3 can be found in biological materials. Saponification is a method commonly used to remove lipids during the extraction of vitamin D from food sources [43, 44]. Furthermore, it has also been reported that protein precipitation and saponification are widely employed in sample pretreatment to facilitate the release of vitamin D and its structural analogs from complex matrices, as well as to eliminate interfering substances.

Upon identifying the optimal *HPLC* conditions for the separation of vitamins D2 and D3, the methodology was applied to the analysis of real egg samples. Figure 2C illustrates the chromatogram of the extract from egg yolk utilized as a test sample, wherein only vitamin D3 was detected. To evaluate the selectivity of the method within the relevant matrix, several extracts derived from different egg yolks were spiked with a standard solution containing both vitamin D2 and D3. A complete separation of the two peaks was consistently observed, as depicted in Figure 2D, which presents the chromatogram obtained from an exemplary real sample.

**FIGURE 3:**
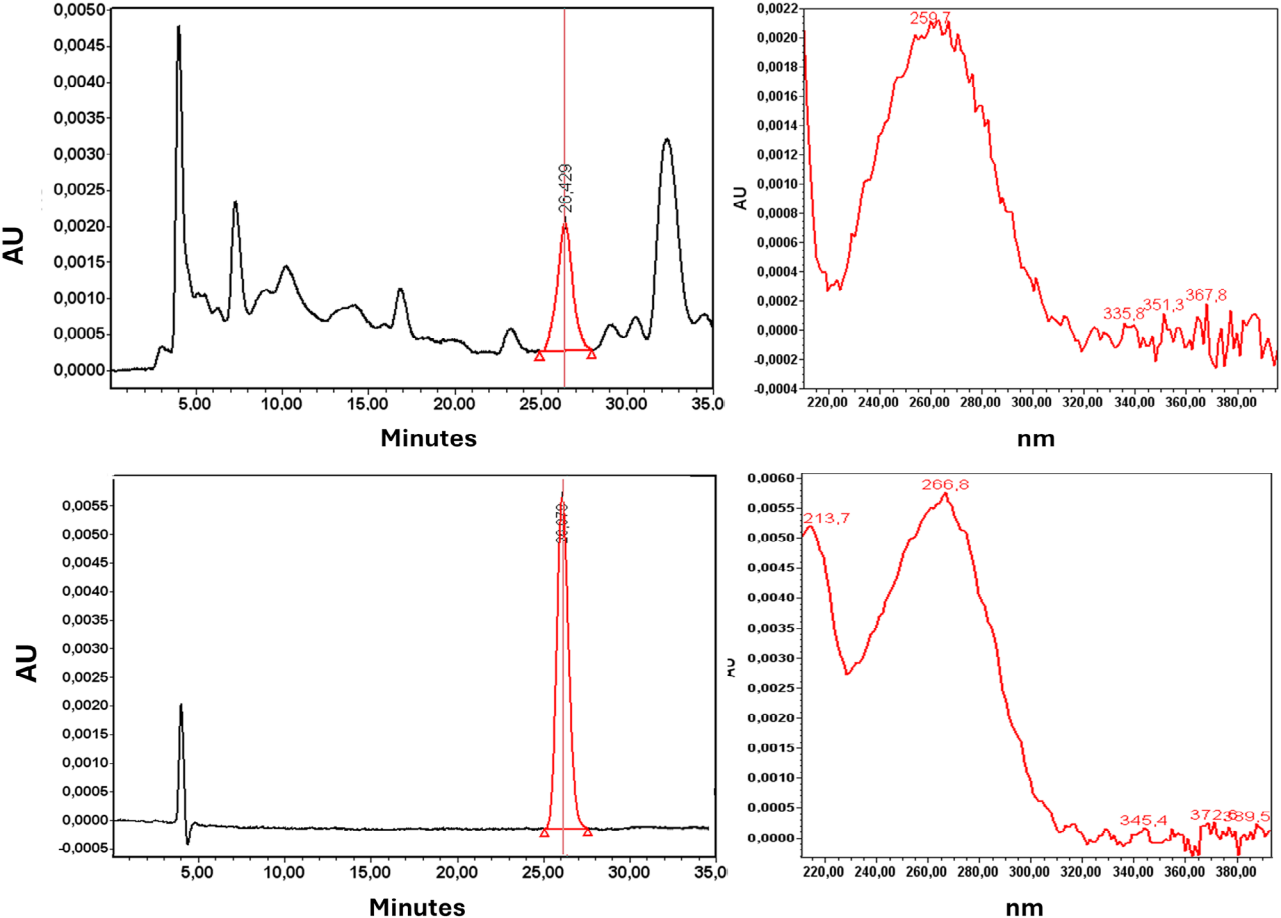
(A) Chromatogram of a real egg yolk sample with the UV spectra of the peak at about 26.429 min; (B) chromatogram of the vitamin D3 standard with the UV spectrum of the peak. The slight difference in retention is due to the matrix effect in the real sample.

To confirm the identity of the vitamin D3 peak in the real sample—which was previously inferred by comparing its retention time with that of the commercial standard—the UV‐Vis spectrum of the vitamin D3 peak from the analysis of the standard was compared with the spectrum of the peak at the corresponding retention time obtained during the analysis of the extracts. This procedure was conducted using the PDA detector coupled to the HPLC system. As clearly shown in Figure 3, a significant correspondence between the UV‐Vis spectrums of the peak at 26.43 min from the analysis of a selected real sample (Figure 3A) and that of the commercial standard (Figure 3B) was observed.

The absence of co‐elution of vitamin D3 with other species is evidenced by the consistent UV spectrum profile across the entire peak width (Figure S2), whereas differing spectral profiles are observed for the neighboring peaks (Figure S3).

The method employed to confirm the identity of the vitamin D3 peak observed in the NARP‐HPLC analysis involved a comprehensive approach that integrates both liquid chromatography and GC–MS. After manually collecting the peak corresponding to the vitamin D3 at approximately 26 min, the sample underwent evaporation to yield a solid form. This solid was subject to a derivatization reaction, an essential step designed to enhance the stability of the vitamin D structures and prevent potential mischaracterization during analysis, consistent with previous findings [45].

**FIGURE 4:**
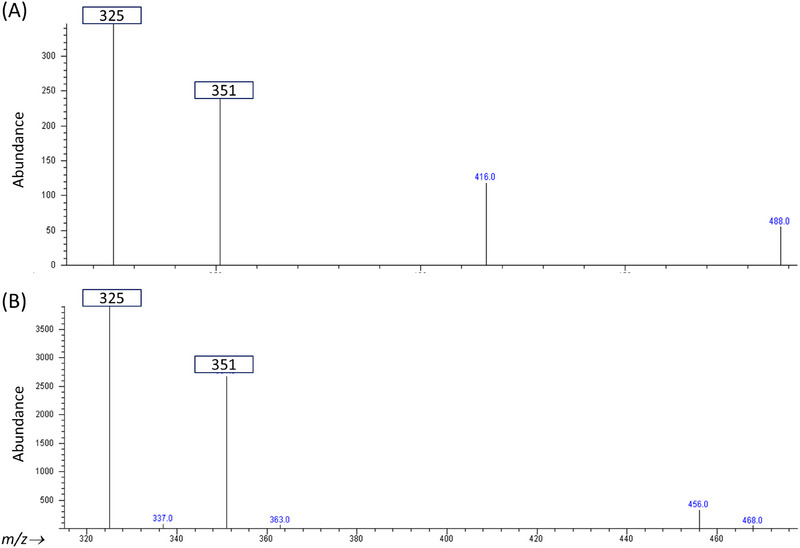
GC–MS spectrum of (A) the standard solution of vitamin D3; (B) the collected peak from the HPLC real sample analysis.

The subsequent GC–MS analysis indicated a strong correlation between the collected NARP‐HPLC peak and the standard vitamin D3 compound, evidenced by matching key parameters: both the base peak and a characteristic ion were identified at *m*/*z* 325 and *m*/*z* 351, respectively (Figure 4). Moreover, the retention time recorded during the GC–MS analysis was consistent with that of the standard vitamin D3, providing further validation. In addition, the relative abundance ratio of the characteristic ions (325–351) was maintained at 1.2, mirroring the ratio observed in the standard reference.

These results collectively substantiate the identification of the peak eluting at around 26 min during the NARP‐HPLC run as vitamin D3, yielding a reasonable degree of confidence in this conclusion.

### 3.3 NARP‐HPLC‐UV Method Validation

As the final phase of the study, the developed NARP‐HPLC‐UV method underwent validation prior to conducting the quantitative analysis of the samples under investigation. *A partial validation* [46, 47, 48, 49] *was carried out* to ensure the method's sufficiency for the objectives of this study. The following parameters were evaluated: selectivity, linearity, precision (short‐term and long‐term), accuracy (short‐term and long‐term), and limit of quantification (LOQ).

Linearity was assessed by constructing a calibration curve, and plotting the concentrations of five calibration standard solutions against their corresponding peak areas. The tested concentration range for linearity was established at 0.2–2 µg/mL, based on preliminary analyses of several egg yolk extracts. The coefficient of determination (*R* ^2^) was found to be 0.9997.

Method specificity was assessed through multiple approaches: spiking egg yolk extracts with vitamin D2 and D3 standards, confirming no co‐elution or interference at the retention time of vitamin D3, analyzing UV spectra across the peak width to ensure purity and homogeneity, and confirming the identity of the vitamin D3 peak through GC–MS, with retention time and mass spectra matching those of the standard. To assess the accuracy and precision of the developed HPLC‐UV method, an external set of two control solutions prepared by spiking egg yolk extracts with known concentrations of vitamin D3 (selected near the extremes of the established calibration points) was utilized: 0.25 and 1.7 µg/mL. Accuracy was assessed by calculating recovery values as the ratio of measured to spiked concentrations, while precision was determined as the relative standard deviation (RSD%) among the concentration values obtained from consecutive injections for both short‐term and long‐term assessments (Table 2). The same experimental protocol was applied to determine the accuracy of the method, expressed as recovery (%). Data presented in Table 2 refer to three replicates of each external test solution conducted within 1 day and over 3 consecutive days.

**TABLE 2:** Intraday and inter‐day RSD% and recovery% values for the analysis of vitamin D3.

Compound	Theoretical conc. (mg/mL)	Intraday precision (RSD%)	Intraday accuracy (recovery%)	Inter‐day precision (RSD%)	Inter‐day accuracy (recovery%)
**Vitamin D3**	0.00025	0.32	101.21	1.09	101.68
		1.36	102.25		
		4.63	103.76		
	0.0017	0.20	102.70	0.69	102.55
		0.51	103.02		
		0.84	101.93		

As indicated by the data presented in Table 2, satisfactory recovery percentages (101.68% and 102.55%) were achieved during the long‐term (inter‐day) evaluation, with RSD% values remaining below 5%. The LOQ was determined based on the lowest concentration at which the RSD% of the peak area values from five consecutive injections was less than 5.0%. *While a 20% limit with an S/N of nine is commonly recommended by guidelines, the stricter 5% limit was chosen to reflect the high precision of the developed method*. Consequently, a LOQ of 0.125 µg/mL was established.

The quantitative analysis of real samples demonstrated an average concentration of vitamin D3 of approximately 4–5 µg/g of egg yolk (wet weight). This assessment accounted for a weight loss of approximately *50%* due to lyophilization. These findings are consistent with the results obtained in other studies [24, 49–52].

## 4 Concluding Remarks

The primary objective of this study was to develop a cost‐effective and easy‐to‐set‐up HPLC method for the accurate quantification of vitamin D3 in egg yolk samples, even in the presence of vitamin D2, *without any preliminary chromatography‐based purification procedure*. Initially, the optimization of HPLC analytical conditions was performed to achieve baseline separation between the vitamin D3 and vitamin D2 peaks, which may either be inherent to the yolk samples undergoing chromatographic analysis or added as an internal standard for the accurate quantification of vitamin D3.

Utilizing predominantly nonaqueous mobile phases, the performance of three distinct C18 stationary phases—each characterized by unique structural properties—was comparatively evaluated. The Xbridge C18 stationary phase exhibited the most favorable performance, prompting further optimization of the following experimental conditions: mobile phase composition, column temperature, and mobile phase flow rate. These optimization efforts resulted in an *R*_S_ of 1.6. This resolution was achieved under the following experimental conditions: a mobile phase consisting of ACN, MeOH, and TFA in a (99/1/0.1, v/v/v) ratio; a column temperature set at 15°C; and a flow rate of 0.5 mL/min.

The developed method was validated following a research‐based protocol, demonstrating satisfactory linearity, precision, accuracy, LOQ, and robustness. Subsequently, the NARP‐HPLC‐UV method was successfully applied to the analysis of real egg yolk samples.

Moreover, the developed HPLC method is easily adapted for use with HPLC‐MS systems, allowing *for further sensitivity enhancements*. This makes the method suitable for a wide range of laboratory settings. By achieving these goals, this study aims to provide a robust and efficient analytical tool for assessing vitamin D levels in food matrices, particularly in egg yolks.

## Author Contributions

**Ina Varfaj**: formal analysis, investigation, methodology, writing–original draft. **Alice Cartoni Mancinelli**: investigation, methodology, writing–review and editing. **Anna Migni**: investigation. **Laura Mercolini**: writing–review and editing. **Cesare Castellini**: writing–review and editing. **Francesco Galli**: writing–review and editing. **Desirée Bartolini**: conceptualization, supervision, writing–review and editing. **Roccaldo Sardella**: conceptualization, supervision, writing–review and editing

## Ethics Statement

The authors hereby affirm that the content of this manuscript has not been published in whole or in part elsewhere. The authors hereby confirm that no animal research has been conducted in this work. The authors hereby declare that they have no conflicts of interest. They affirm that there are no known competing financial interests or personal relationships that could be perceived as influencing the work presented in this paper.

## Conflicts of Interest

The authors declare no conflicts of interest.

## Supporting information



Supporting Information

## Data Availability

The data that support the findings of this study are available on request from the corresponding author. The data are not publicly available due to privacy or ethical restrictions.
