# Supplementary material for: A Cost‐Effective Nonaqueous Reversed‐Phase High‐Performance Liquid Chromatography Method to Measure Vitamin D3 in Hen's Egg Yolk
Source: J Sep Sci. 2025 Jan 23;48(1):e70087. doi: 10.1002/jssc.70087 (PMC11755696; doi:10.1002/jssc.70087)
Supplement: Supplementary file 1 — Supporting Information [file JSSC-48-e70087-s001.docx]

**SUPPORTING INFORMATION**

**A cost-effective non-aqueous reversed-phase high-performance liquid chromatography method to measure vitamin D3 in hens egg yolk**

Ina Varfaj^1^, Alice Cartoni Mancinelli^2^, Anna Migni^1^, Laura Mercolini^3^, Cesare Castellini^2^,

Francesco Galli^1^, Desirée Bartolini^1^*, Roccaldo Sardella^1^*

^1^Department of Pharmaceutical Sciences, University of Perugia, Via del Giochetto, 06122 Perugia, Italy

^2^Department of Agricultural, Food and Environmental Sciences, University of Perugia, Borgo XX Giugno 74, 06100 Perugia, Italy

^3^Department of Pharmacy and Biotechnology (FaBiT), Alma Mater Studiorum - University of Bologna, Via Belmeloro 6, 40126 Bologna, Italy

Corresponding authors: Desirée Bartolini (desiree.bartolini@unipg.it) and Roccaldo Sardella (roccaldo.sardella@unipg.it)


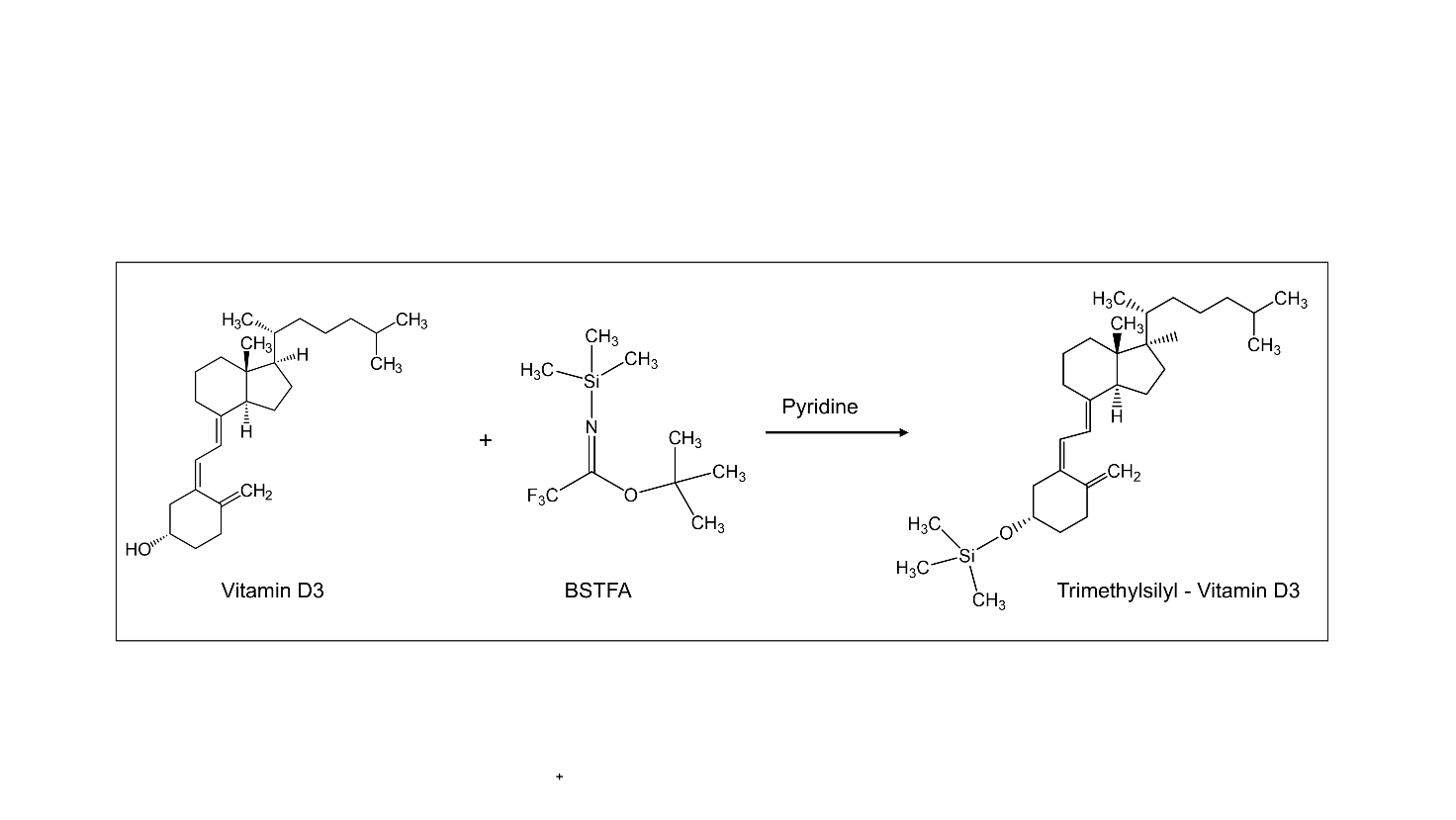


**Figure S1.** The reaction scheme for vitamin D3 derivatization is shown.


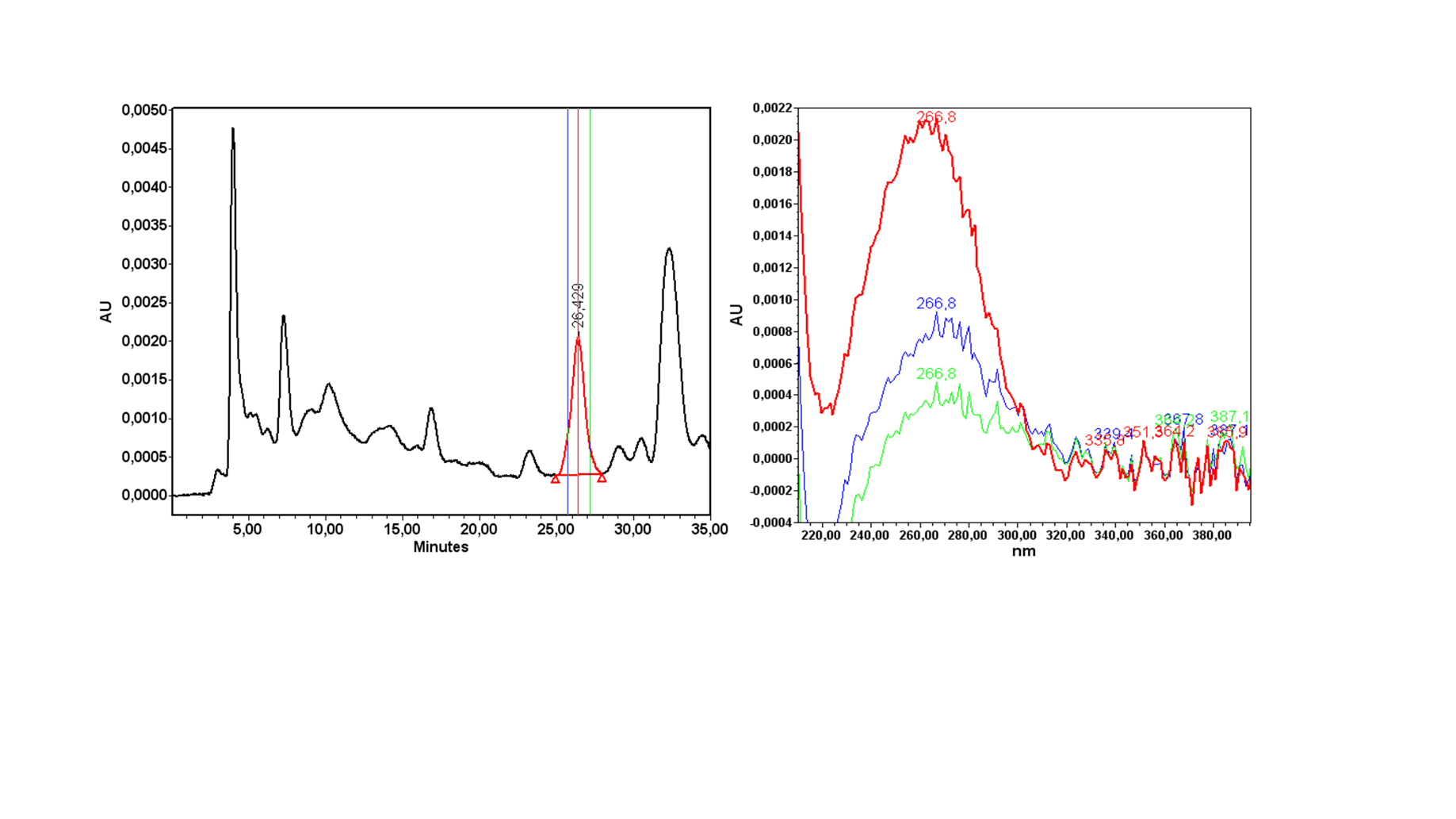


**Figure S2.** Selected time-window of the chromatogram of a real egg yolk sample with the UV spectra of the peak at about 26.429 min (vitamin D3 peak), recorded in three different points of the peak.


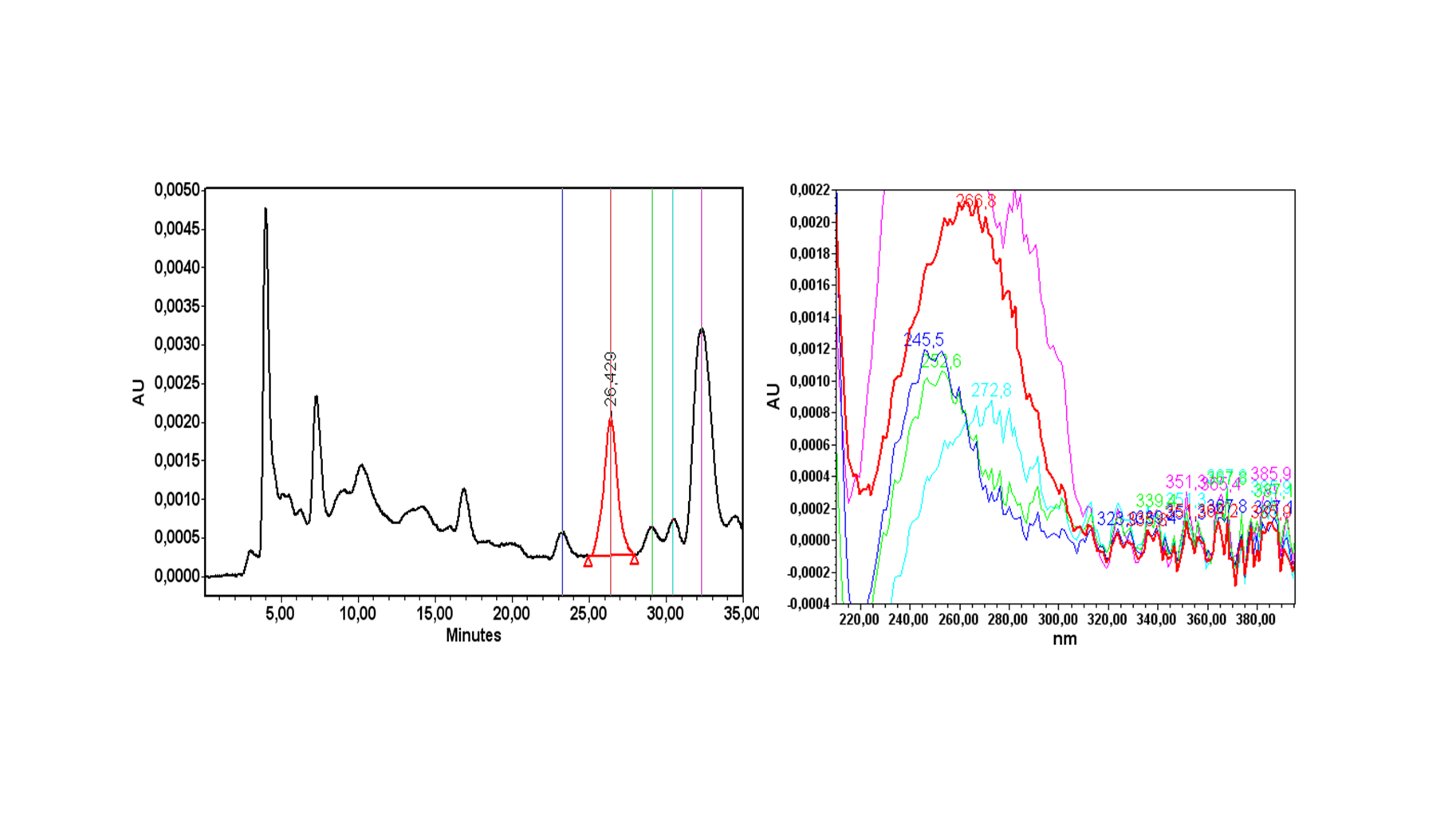


**Figure S3.** Chromatogram of a real egg yolk sample with the UV spectra of the peak at about 26.429 min (vitamin D3 peak), and that of its neighbouring peaks.
